# Sinonasal osteosarcoma of the ethmoid bone with decade-later recurrence presenting as early orbital apex syndrome: an 18-year case report

**DOI:** 10.1097/RC9.0000000000000841

**Published:** 2026-08-24

**Authors:** Muhammad Ehtesham, Hamza Khan Khattak, Maryam Shoaib, Imran Khan, Azam Nawaz, Kamil Ahmad Kamil

**Affiliations:** a Department of Medicine, Khyber Teaching Hospital, Peshawar, Khyber Pakhtunkhwa, Pakistan; b Department of ENT, Khyber Teaching Hospital, Peshawar, Khyber Pakhtunkhwa, Pakistan; c Internal Medicine Department, Nasrat Bashir Curative Hospital, Kandahar, Afghanistan

**Keywords:** case report, endoscopic endonasal surgery, orbital apex syndrome, paranasal sinus neoplasms, sinonasal osteosarcoma

## Abstract

**Introduction:**

Primary ethmoid osteosarcoma is an exceptionally rare craniofacial malignancy, characterized by aggressive local invasion of adjacent structures. While most recurrences occur within the first 2–3 years, late relapse after long-term disease-free survival is exceedingly uncommon.

**Case presentation:**

We describe an 18-year clinical course of a patient first diagnosed with ethmoidal chondroblastic osteosarcoma in childhood. He was treated with chemotherapy and radiation therapy and went into remission. Seven years later, the cancer recurred in the sinuses and lungs. He underwent surgery and more chemotherapy, then remained stable for nearly a decade. After that, he developed worsening nasal blockage, nosebleeds, eye bulging, and sudden vision loss consistent with orbital apex syndrome. Scans showed a destructive mass eroding the skull base and reaching the orbital apex. He underwent endoscopic surgery to remove the tumor, decompress the optic canal, and reconstruct the skull base. Pathology confirmed another recurrence. After surgery, his vision improved to light perception, and he started a new round of chemotherapy.

**Clinical discussion:**

This case shows that sinonasal osteosarcoma can recur very late, so lifelong monitoring with CT and MRI is necessary. Sudden ocular symptoms in these patients should be treated as emergencies to preserve vision. An endoscopic approach allowed safe access to the skull base and effective orbital decompression.

**Conclusion:**

Timely multidisciplinary care and continued follow-up are essential for rare malignancies, even after many years without disease.

## Introduction

Primary osteosarcoma of the craniofacial skeleton is rare (~1% of head and neck malignancies), with ethmoid involvement being far less common than the mandible and maxilla^[^1,2^]^. Unlike long-bone osteosarcomas, it typically affects younger adults^[^1,2^]^ and spreads mainly by local invasion rather than metastasis, a factor that determines its prognosis^[^1–3^]^. Surgical resection with negative margins is the main treatment^[^2,4^]^, but it is often limited by nearby neurovascular and orbital structures, leading to high recurrence rates^[^1,5^]^. While a 5-year disease-free interval is often considered curative in osteosarcoma^[^6–8^]^, craniofacial tumors may recur late, though this is rare^[^2,6–9^]^. We report an 18-year course culminating in very late sinonasal osteosarcoma recurrence presenting with orbital apex syndrome and vision loss, highlighting the importance of early recognition and timely multidisciplinary management. This case report is presented in accordance with the SCARE checklist[10].

HIGHLIGHTS

- Primary osteosarcoma of the ethmoid bone is an exceptionally rare craniofacial malignancy.
- The case demonstrates a prolonged 18-year clinical course with two delayed recurrences.
- Very late recurrence occurred after an extended disease-free interval, which is uncommon in osteosarcoma.
- Recurrence presented as early orbital apex syndrome with acute optic nerve compression.
- Endoscopic endonasal resection enabled effective decompression with reduced surgical morbidity compared to prior open surgery.

## Case presentation

A 33 year-old male scrap collector from Afghanistan presented with a 3-month history of progressive left nasal obstruction and facial fullness, associated with intermittent epistaxis, headache, and non-rotatory dizziness. Over the past few days, his condition had worsened, with rapidly progressive left-sided proptosis followed by sudden, painless vision loss in the left eye. He denied fever, trauma, infectious symptoms, or drug use. Four months earlier, he had undergone left cataract surgery, with satisfactory postoperative vision and no evidence of proptosis at the time. His medical history was significant for chronic hepatitis C infection, likely acquired perinatally, and a complicated history of sinus osteosarcoma.

His past oncologic history was extensive and relevant to his current presentation. At the age of 12 years, he was diagnosed with a malignant sinonasal tumor extending toward the orbit and intracranial compartment, reported as osteosarcoma with chondroid differentiation. According to the patient, he believed that his condition might have originated after a childhood nasal penetration caused by a Keekar (Acacia nilotica) thorn; however, this association was purely speculative. He subsequently underwent 24 doses of multi-agent chemotherapy (cisplatin, methotrexate, doxorubicin) and 30 fractions of radiotherapy, which achieved initial remission, as documented in contemporaneous records. However, 7 years later, the patient developed a local recurrence in the left sinonasal region with concurrent pulmonary metastases. He again underwent gross total resection of the sinonasal mass via an open lateral rhinotomy, while pulmonary metastases were managed with systemic chemotherapy. He required prolonged inpatient care followed by regular oncology follow-up and remained clinically and radiologically disease-free for approximately the next ten years.

**Figure 1.**
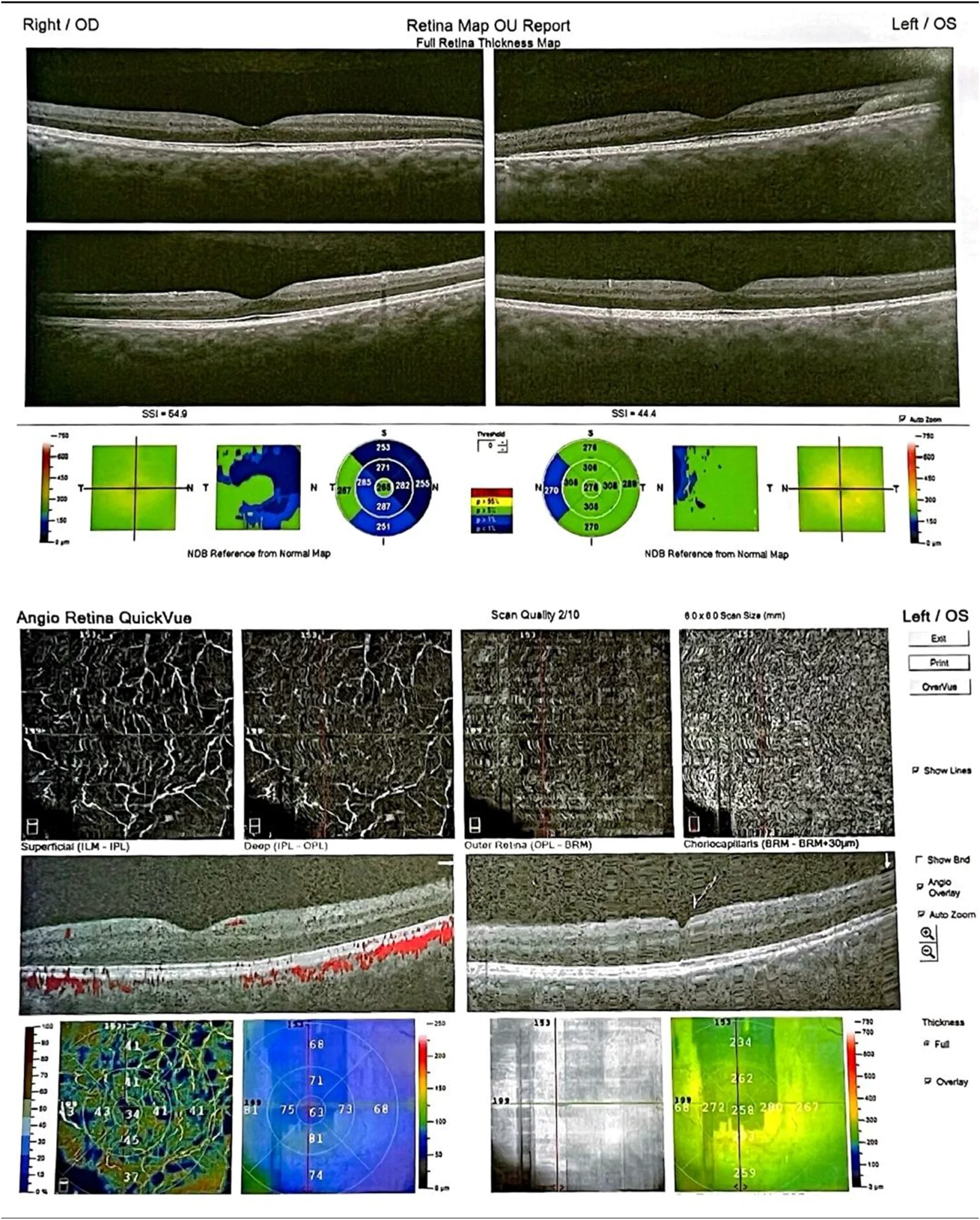
Multi-modal optical coherence tomography (OCT) of the left eye. (Above) The full retinal thickness map (SSI 44.4) shows no evidence of significant macular edema, atrophy, or other primary retinal pathology. Thickness values fall within normative limits. (Below) Corresponding OCT angiography (OCTA) of the macula was obtained using the AngioVue system with software (Version 2018.0.0.18; Optovue, Inc., Fremont, CA, USA). En face angiograms of the superficial capillary plexus (SCP), deep capillary plexus (DCP), outer retina, and choriocapillaris reveal intact vascular arcades without evidence of capillary non-perfusion, microvascular abnormalities, or macular neovascularization. The absence of structural or vascular pathology supports a retrobulbar or prechiasmal localization of the lesion (i.e., Compressive optic neuropathy) as the etiology of the patient’s complete loss of light perception.

On examination during the current admission, facial asymmetry and left-sided proptosis were obvious. There was no cervical lymphadenopathy. Ophthalmologic evaluation demonstrated complete visual loss in the left eye with no perception of light (NPL) and a relative afferent pupillary defect. There was mild restriction noted during abduction in the left eye on extraocular movement; other cranial nerves were intact. Nasal endoscopy revealed a large mass occupying the left nasal cavity with deviation of the nasal septum toward the right. Optical coherence tomography of the retina (Fig. 1) showed no retinal or macular pathology, suggesting a retrobulbar cause of vision loss. Routine labs, including liver function tests, were normal except for a weakly positive hepatitis C serology (Table 1).

**Table 1:** Laboratory and diagnostic findings of the patient.

Parameter	Observed value	Normal range	Parameter	Observed value	Normal range
Hematology			Biochemistry		
(a) Complete blood count (CBC)			(a) Renal function tests (RFTs)		
WBC	11 800 /µL	4000–11 000 /µL	Urea	22 mg/dL	15–50 mg/dL
RBC	5.08× 10^6^/µL	4.5–5.5× 10^6^/µL	Creatinine	0.8 mg/dL	0.5–1.3 mg/dL
Hemoglobin	15.4 g/dL	13.8–17.2 g/dL	Uric acid	3.7 mg/dL	3.6–5.7 mg/dL
HCT	42.1 %	40–50 %	eGFR	126.45 mL/min	>60 mL/min
(b) Red cell indices			(b) Liver function tests (LFTs)		
MCV	82.9 fL	80–100 fL	SGPT (ALT)	31 U/L	<45 U/L
MCH	30.3 pg	27–32 pg	Bilirubin total	0.9 mg/dL	0.2–1.0 mg/dL
MCHC	36.5 g/dL	32–36 g/dL	Alkaline Phophatase	214 U/L	65–240 U/L
RDW-CV	14.7 %	11.5–14.5 %	(c) Glycemic profile		
(c) Platelet indices			Random blood sugar	90 mg/dL	80–150 mg/dL
Platelets	319 000 /µL	150 000–400 000 /µL	Serology		
MPV	8.9 fL	7.2–11.1 fL	HBsAg (by ICT)	Negative	Non-reactive
PDW	15.7 %	9.0–17.0 %	Anti-HCV (by ICT)	Weak positive	Non-reactive
PCT	0.28 %	0.10–0.50 %	Anti-HIV (by ICT)	Negative	Non-reactive
(d) White cell count			Ocular findings (at 4 months pre-op)		
Neutrophils	66%	40–75%	Visual acuity (R)	6/60	6/6 (Normal)
Lymphocytes	26%	20–45%	Visual acuity (L)	6/9	6/6 (Normal)
Monocytes	06%	02–10%	IOP (L)	18 mm Hg	10–21 mm Hg
Eosinophils	02%	00–06%	IOP (R)	20 mm Hg	10–21 mm Hg
(e) Coagulation profile			Axial length (A-scan) (L)	22.98 mm	
PT patient	13 sec	12 sec	Corneal curvature (K1/K2) (L)	46.50/45.00 D	D ~ 44.00 D
APTT patient	32 sec	30 sec	ACD (anterior chamber depth) (L)	3.10 mm	2.5–4.0 mm
INR	1.09	–	Lens (L)	PSC (posterior subcapsular cataract)	

**Figure 2.**
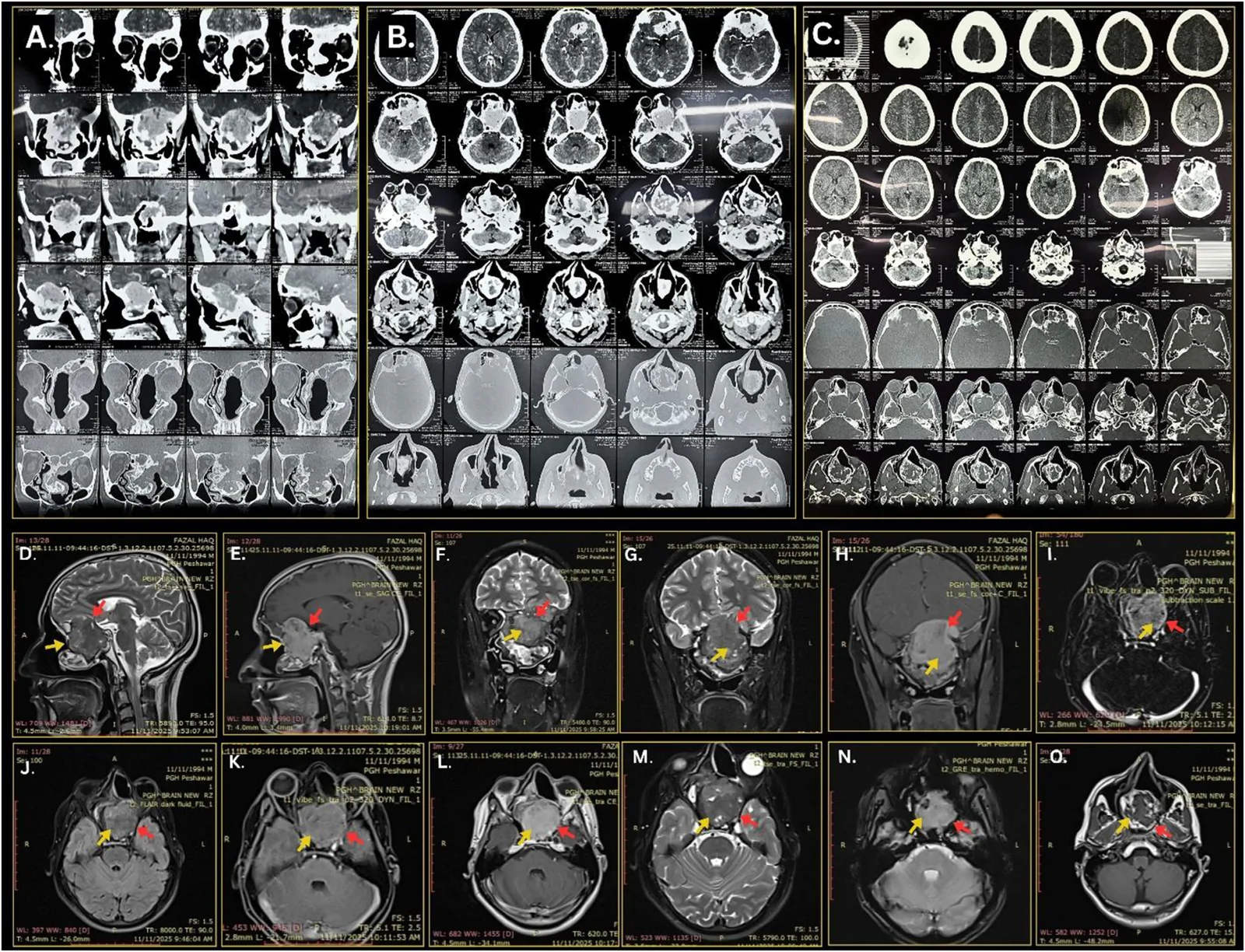
Cross-sectional imaging: (**A–C**) Contrast-enhanced CT (axial, coronal, sagittal) shows a large expansile, destructive lesion (5.3 × 5.3 × 5.5 cm) arising from the nasal septum and left ethmoid air cells, eroding the left lamina papyracea, extending to the left orbital apex with optic nerve compression and superiorly into the frontal bone and pituitary fossa, with epidural extension abutting the left frontal lobe. The right orbit and optic nerve are intact. (**D–O**) Multiplanar contrast-enhanced MRI depicts the tumor (yellow arrow), whereas the red arrows indicate the optic nerve. Imaging characterizes a 6.0 × 5.0 × 4.6 cm enhancing soft-tissue mass in the posterior left nasal cavity with marked rightward septal deviation, extending into the nasopharynx, sphenoid sinus, and sellar region with dural enhancement (meningeal tail). Critical involvement includes mass effect on the left optic nerve and abutment of the left internal carotid artery, without definitive vascular invasion.

Imaging via contrast-enhanced CT (Fig. 2A–C) and MRI (Fig. 2D–O) showed a large destructive sinonasal mass centered in the left ethmoid region with skull base and orbital apex extension, indicating early orbital apex syndrome. The lesion exerted significant mass effect on the left optic nerve and closely abutted the left internal carotid artery without definitive radiological evidence of vascular invasion. The rest of the brain parenchyma, optic chiasm, pituitary gland, brainstem, cranial nerves (including the trigeminal and vestibulocochlear nerves), and intracranial vessels showed no abnormalities. Based on the clinical history and imaging findings, a provisional diagnosis of late recurrent sinonasal osteosarcoma was made.

**Figure 3.**
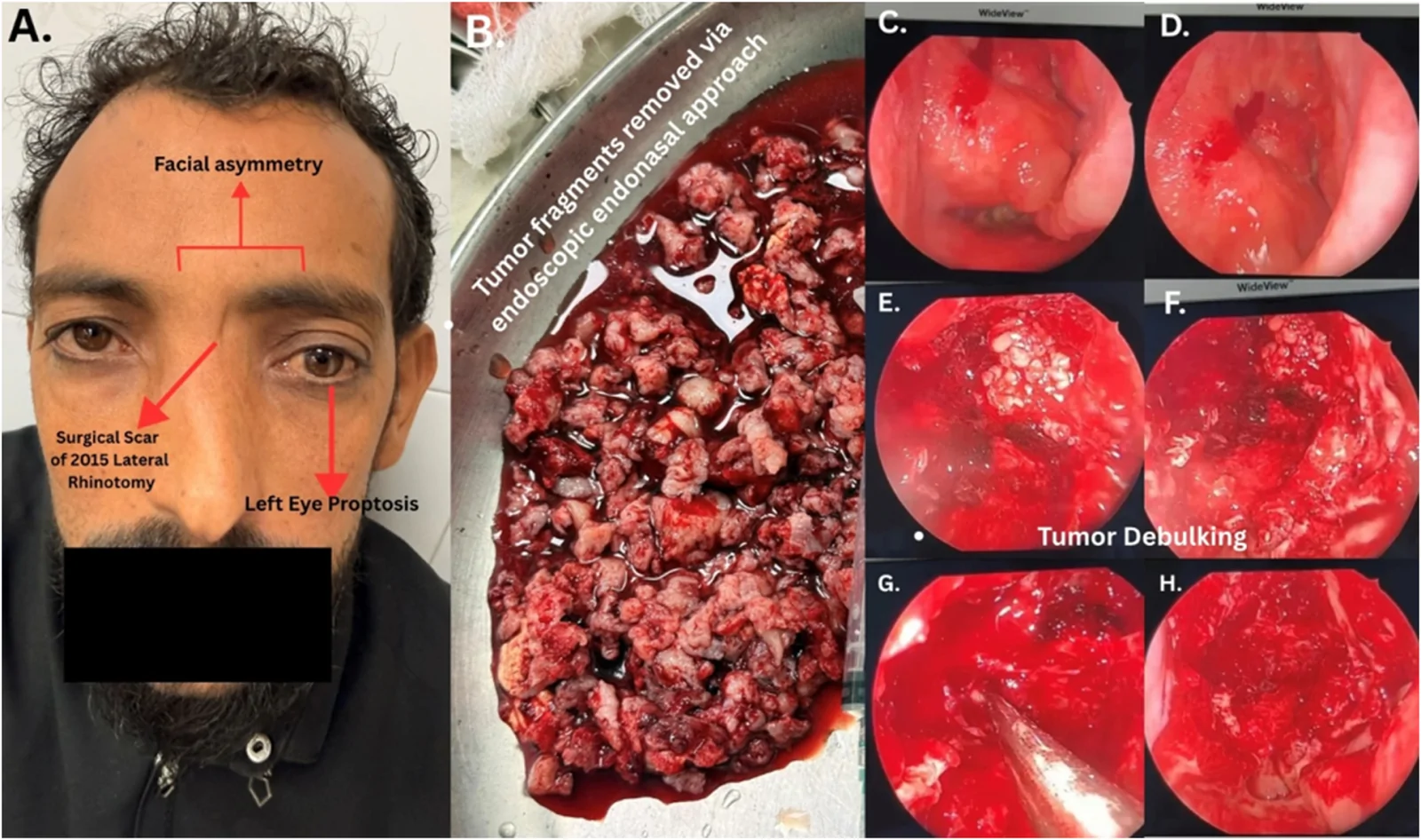
(A) Clinical presentation, surgical specimen, and intraoperative endoscopic views. (B) Gross appearance of multiple tumor fragments showing irregular, hemorrhagic tissue consistent with a sinonasal tumor. (C–H) Intraoperative endoscopic views of the sinonasal cavity demonstrating a friable, highly vascular tumor in the ethmoid region, with areas of bleeding. Sequential images show the progressive endoscopic tumor debulking and removal, enabling decompression of the sinonasal compartment and improving access toward the orbital apex to relieve optic nerve compression.

The case was reviewed in a multidisciplinary tumor board (ENT, neurosurgery, ophthalmology, radiology, oncology), and surgical management was agreed upon. The patient subsequently underwent endoscopic endonasal resection with neurosurgical assistance for maximal safe debulking and skull base clearance (Fig. 3). During the procedure, the optic canal was decompressed and, as no gross dural invasion was seen, dural resection was not required. A CSF leak was encountered during skull base clearance and repaired using a multilayer closure technique with an underlay graft. Skull base reconstruction was performed using a pedicled nasoseptal (Hadad–Bassagasteguy) flap, providing vascularized coverage of the skull base defect. The procedure lasted 5 hours and was completed without complications.

Postoperatively, the patient received antibiotics, corticosteroids, analgesics, and nasal care protocols. Vision in the left eye improved to NPL only, but no further visual function was regained. He was discharged after one week with ENT follow-up planned. Histopathology of the resected tumor revealed a high-grade malignant mesenchymal neoplasm composed of pleomorphic spindle-to-polygonal cells arranged in sheets and fascicles, with areas of malignant osteoid and atypical mitoses, confirming recurrent osteosarcoma. Following recovery from surgery, the patient was started on adjuvant systemic chemotherapy. Postoperatively, nasal crusting occurred and was managed conservatively with saline irrigation and endoscopic debridement (Clavien–Dindo grade I complication requiring no pharmacological or surgical intervention).

The current regimen consists of doxorubicin, cisplatin, and high-dose methotrexate planned for six cycles, adjusted based on response. Despite weakly positive hepatitis C serology, persistently normal LFTs permitted methotrexate use with close monitoring. At the time of writing, the patient is undergoing chemotherapy and remains under close oncological surveillance. He is clinically stable, with only NPL recovered in the left eye, and otherwise maintains functional status and regular follow-up.

## Discussion

The present case is notable because the tumor arose from the ethmoid bone, a rare primary site in contrast to previously reported skull-base osteosarcomas predominantly arising from the sphenoid sinus, clivus, or sellar region^[^2,11–13^]^, and the patient demonstrated two exceptionally late recurrences over a period of 18 years. This case challenges the conventional 5-year disease-free survival benchmark often considered indicative of cure in systemic malignancies, with the patient developing a first recurrence 7 years after initial treatment and a second recurrence after an additional decade of disease stability[9]. Furthermore, progression from nasal obstruction and epistaxis to unilateral proptosis and acute painless vision loss illustrates the severe functional consequences of local tumor extension. Unlike previously reported cases that described favorable visual recovery after decompression, recovery in our patient remained limited, likely due to delayed presentation. The patient’s posterior subcapsular cataract, operated on 4 months earlier, likely represents a delayed radiation-induced effect from childhood therapy rather than a direct tumor effect[14].

The confined anatomy of the sinonasal compartment predisposes osteosarcomas to aggressive local invasion with orbital and skull-base extension ^[^1,5^]^. Extension into the orbital apex or optic canal may produce orbital apex syndrome and compressive or ischemic optic neuropathy[5] as observed in this case. Orbital apex syndrome in sinonasal malignancies varies according to the extent of apical invasion. Although our tumor extended into the orbital apex and optic canal, it presented as an early or partial form characterized by isolated optic nerve dysfunction, whereas more advanced disease may involve additional cranial nerves[15]. Importantly, vascular planes remained preserved despite proximity to the internal carotid artery, facilitating safer endoscopic decompression. Advanced skull-base involvement may be associated with significant morbidity and can limit the feasibility of radical resection^[^3,4,11^]^.

In patients with a history of head and neck malignancies, recurrence should be considered early in the differential diagnosis. Nonspecific symptoms such as nasal obstruction, facial fullness, and intermittent epistaxis may mimic benign conditions, delaying diagnosis^[2,11]^. Prior oncologic treatment may further complicate assessment by producing fibrosis and anatomic distortion that resemble recurrent disease[1]. In the present case, diagnostic considerations included late recurrence, radiation-induced secondary osteosarcoma, and post-treatment changes. The lesion arose in the same sinonasal region as the primary tumor, and histopathology confirmed recurrent chondroblastic osteosarcoma, favoring late recurrence over radiation-induced malignancy. Moreover, the aggressive skull-base erosion and orbital apex compression argued against non-expansile post-treatment entities such as osteoradionecrosis or chronic radiation-induced rhinosinusitis. Cross-sectional imaging played a crucial role, with CT demonstrating cortical destruction and matrix mineralization, while MRI better defined orbital and intracranial extension for staging and surgical planning^[^2,11^].^ Other aggressive sinonasal malignancies were excluded by the identification of malignant osteoid formation^[^1,16,17^]^.

Management of sinonasal osteosarcoma typically combines maximal safe resection with adjuvant chemotherapy, while radiotherapy is reserved for unresectable disease, positive margins, or high-risk features^[^1,2,4^].^ There is a lack of large prospective studies to guide standardized treatment due to the rarity of the disease[18]. In the present case, an endoscopic endonasal tumor debulking and decompression of critical structures, including the optic nerve, was performed, while minimizing morbidity associated with open craniofacial resection. Compared with the 2015 open lateral rhinotomy, the current endoscopic endonasal approach offers similar tumor control with reduced morbidity, better orbital preservation, and improved functional and cosmetic outcomes[5]. In our case, intraoperative cerebrospinal fluid leakage requiring multilayer repair parallels skull-base challenges described in post-treatment cases by Yamada et al[19].

Adjuvant chemotherapy consisting of high-dose methotrexate, doxorubicin, and cisplatin was administered following surgery, consistent with established MAP-based protocols for high-grade osteosarcoma[14]. The decision to prioritize surgical debulking was driven by the acute onset of orbital apex syndrome and the need for urgent optic nerve decompression, while further radiotherapy was limited by prior radiation exposure and concerns regarding cumulative toxicity to adjacent critical structures. Despite timely intervention, visual recovery remained incomplete, likely reflecting the duration and severity of preoperative optic nerve compromise^[^5,11^]^.

Several key learning points emerge from this case. First, very late recurrence of sinonasal osteosarcoma, although uncommon, remains possible and should be considered when new sinonasal symptoms arise, even after prolonged disease-free intervals. Second, orbital involvement and optic nerve compression may result in irreversible visual deficits despite surgical and adjuvant treatment. Third, long-term surveillance strategies may need to extend beyond traditional follow-up periods in selected high-risk patients. Strengths of this report include a well-documented clinical history and prompt multidisciplinary management involving ophthalmology, oncology, otorhinolaryngology, and neurosurgery. However, certain limitations should be acknowledged. Original histopathological material from the primary childhood tumor was unavailable, precluding direct comparison with the recurrent lesion, and the absence of molecular or genetic profiling limited deeper biological interpretation.

## Conclusion

This case highlights the unpredictable behavior of sinonasal osteosarcoma, which may recur after long disease-free intervals and present with aggressive skull-base extension causing severe neuro-ophthalmic deficits. This challenges the adequacy of 5-year follow-up and supports prolonged surveillance. Furthermore, rapidly progressive vision loss in patients with previously treated malignancy should prompt urgent assessment of orbital apex involvement, as early decompression may be the only chance for visual preservation. Finally, management requires a multidisciplinary approach, with endoscopic endonasal surgery enabling safe decompression and debulking while minimizing morbidity in anatomically complex post-treatment cases.

## Data Availability

The data supporting the findings of this study are available from the corresponding author upon reasonable request. Data are not publicly available due to patient privacy and confidentiality considerations.

## Patient’s perspective

As a child, I underwent repeated treatments for a nasal tumor, which were physically and emotionally challenging. Years later, when sudden vision loss occurred, I felt anxious and uncertain about my health. Undergoing surgery and chemotherapy again was difficult, but I felt supported by the coordinated care from multiple specialists. Adjusting to vision loss has been challenging, but I am reassured, knowing that I am being closely monitored and receiving ongoing care.

## Artificial intelligence declaration

**Purpose and scope of AI use**: Generative artificial intelligence (ChatGPT) was used solely to assist with language refinement, including grammar correction and sentence structure improvement, during manuscript drafting and revision stages. It was not used for study design, data analysis, image generation, clinical interpretation, or scientific content generation. The authors take full responsibility for the accuracy, integrity, and originality of all content.

**AI Tool(s) and configuration**: ChatGPT (OpenAI; GPT-4 model; accessed in March 2026) was used via a secure cloud-based interface. Default system settings were applied; no plug-ins, fine-tuning, or parameter modifications were used.

**Data inputs and safeguards**: Only anonymized manuscript text was provided to the AI tool. No identifiable patient data, images, or confidential institutional information was shared. All inputs were fully de-identified and compliant with applicable data protection standards.

**Human oversight and verification**: All AI-assisted outputs were critically reviewed, edited, and verified by the authors for clinical accuracy and scientific validity. Any suggestions generated by the tool were subject to manual revision or rejection, as appropriate.

**Bias, ethics, and regulatory compliance**: The authors reviewed all AI-assisted content to ensure the absence of bias or inaccuracies. No conflicts of interest or financial relationships with AI vendors exist.

**Reproducibility and transparency**: Not applicable. Artificial intelligence was used solely for language editing and grammar refinement, and no AI-generated scientific data, code, or analytical outputs were produced.
